# Enhancing the functionality of self-assembled immune signals using chemical crosslinks

**DOI:** 10.3389/fimmu.2023.1079910

**Published:** 2023-02-06

**Authors:** Marian Ackun-Farmmer, Christopher M. Jewell

**Affiliations:** ^1^Fischell Department of Bioengineering, University of Maryland, College Park, MD, United States; ^2^US Department of Veterans Affairs, Veterans Affairs Maryland Health Care System, Baltimore, MD, United States; ^3^Robert E. Fischell Institute for Biomedical Devices, College Park, MD, United States; ^4^Department of Microbiology and Immunology, University of Maryland Medical School, Baltimore, MD, United States; ^5^Marlene and Stewart Greenebaum Cancer Center, Baltimore, MD, United States

**Keywords:** layer-by-layer self-assembly, polyelectrolyte multilayer, cross-linking, microparticle and nanoparticle, vaccine, immunotherapy

## Abstract

Multiple sclerosis (MS) is an autoimmune disease that develops when dysfunctional autoreactive lymphocytes attack the myelin sheath in the central nervous system. There are no cures for MS, and existing treatments are associated with unwanted side effects. One approach for treating MS is presenting distinct immune signals (i.e., self-antigen and immunomodulatory cues) to innate and adaptive immune cells to engage multiple signaling pathways involved in MS. We previously developed immune polyelectrolyte multilayer (iPEM) complexes built through layer-by-layer deposition of self-antigen - myelin oligodendrocyte glycoprotein (MOG) - and toll-like receptor antagonist, GpG to treat MS. Here, glutaraldehyde-mediated stable cross-links were integrated into iPEMs to load multiple classes of therapeutics. These cross-linked iPEMs maintain their immunological features, including the ability of GpG to blunt toll-like-receptor 9 signaling and MOG to expand T cells expressing myelin-specific T cell receptors. Lastly, we show that these functional assemblies can be loaded with a critical class of drug - mTOR inhibitors - associated with inducing regulatory T cells. These studies demonstrate the ability to incorporate small molecule drugs in reinforced self-assembled immune signals juxtaposed at high densities. This precision technology contributes new technologies that could drive antigen-specific immune response by simultaneously modulating innate and adaptive immunity.

## 1 Introduction

Autoimmune diseases occur when immune cells mistakenly recognize and destroy host cells within the body. Multiple sclerosis (MS) is an autoimmune disease that affects over 1 million adults in the US (1). MS is caused by autoreactive lymphocytes (e.g., T, B, and natural killer cells) that cross the blood-brain-barrier and enter the central nervous system to attack the neuronal protective sheath - myelin. Owing to the complex immunopathology of MS, current treatments are non-curative, non-specific, and accompanied with high compliance burden (2, 3). Based on the current clinical unmet needs in treating MS, antigen-specific immunotherapies that offer precision control are urgently needed to selectively modulate the immune dysfunction in MS without the side-effects facing current treatment options.

Impaired immune cells in MS are potential therapeutic targets for treating the disease. Antigen-presenting cells (APCs) such as dendritic cells (DCs) that present antigen and costimulatory signals to drive the differentiation of naïve T cells are altered in MS (4). Thus, emerging therapies aim to reprogram these cells through controlled delivery of immune cues consisting of self-antigens (i.e., myelin oligodendrocyte glycoprotein - MOG) and immunomodulators (i.e., oligonucleotides, small molecule drugs) to treat MS without broad immunosuppression. Toll-like receptors (TLRs) on APCs that recognize pathogen-associated molecular patterns (PAMPs) found on foreign pathogens are overexpressed in human and preclinical MS models. In recent years, TLRs have become attractive targets for treating MS (5–8). Thus, one strategy for treating MS is combining self-antigen with TLR antagonists to downregulate inflammation and promote regulatory T cells (T_REGS_) that suppress disease to induce immune tolerance.

Self-assembled biomaterials that integrate dosing, co-delivery, spatial, and temporal delivery of distinct immune signals are urgently needed to improve MS treatments. These materials exploit naturally occurring processes (i.e., electrostatic interactions, hydrophobic forces, hydrogen bonding, host-guest interactions, bio-specific interactions, and van-der Waals forces) (9) to form ordered assemblies of desired geometries. Of particular interest are polyelectrolyte multilayers (PEMs) built through layer-by-layer (LbL) electrostatic assembly of oppositely charged biomolecules on planar surfaces and colloidal particles. For drug delivery, vaccines, and immunotherapies, PEMs have been developed using synthetic materials (i.e., polymers, inorganic particles) (10, 11) to electrostatically condense a range of biological cargos (i.e., peptides, nucleic acids, protein aggregates) (12).

We recently pioneered tunable immune polyelectrolyte multilayers (iPEMs) composed entirely of disease-relevant antigens and modulatory immune signals built using LbL assembly on planar substrates or sacrificial templates (10, 11, 13–18). Unlike other PEMs, capsular iPEMs are built entirely from immune cues (i.e., self-antigen and oligonucleotides) assembled on sacrificial colloidal calcium carbonate templates, which eliminates intrinsic inflammatory effects associated with many synthetic materials (14, 16, 17, 19). In particular, we previously assembled iPEMs with positively charged disease-relevant myelin oligodendrocyte glycoprotein (MOG) antigen and a negatively charged antagonist of toll-like receptor 9 (TLR9) – GpG. We show in previous work that the high-density juxtaposition of MOG and GpG downregulates DC activation to promote regulatory T cells and induce partial immune tolerance in a preclinical MS model (16, 19).

MOG/GpG iPEMs deliver MOG through major histocompatibility complex (MHC) molecules present on APCs to the T-cell receptors on antigen-specific T cells, while the GpG oligonucleotide blunts TLR9 in the endosome of APCs. By co-delivering MOG and the TLR9 antagonist GpG, we have shown the MOG in these iPEMs drive proliferation of antigen-specific T cells, while the GpG blunts TLR9 signaling to deactivate APCs (16, 19). Towards the goal of driving robust immune tolerance to treat MS, co-delivery of small molecule immunomodulators associated with T_REG_ induction are being actively pursued as a way to target adaptive immune cells (e.g., self-reactive T cells) involved in MS. For example, studies modulating the immune system by delivering self-antigen with rapamycin (Rapa), an mTOR inhibitor can promote T_REGS_ and induce tolerance in preclinical MS models (13, 20–24). Thus, next generation iPEMs integrate new nanotechnology features – such as the ability to load and co-deliver Rapa or other small molecule immune cues could enhance the potency and selectivity of antigen-specific tolerance therapies.

While iPEMs offer the unique benefits to drive tolerance just described, electrostatic interactions used to maintain the assemblies can be weakened in the presence of salts, ions, or other destabilizing environmental conditions (25). Thus, inclusion of facile chemistries such as crosslinking, could provide additional tunability and stability, while creating the capability to load broader classes of cargo, such as small molecule drugs. Formation of cross-links in microcapsules by use of cross-linkers such as glutaraldehyde (GA) has been successfully achieved in other PEM assemblies to modify the permeability of PEMs and achieve drug loading and sustained release (26). While the benefits of GA cross-linking have been demonstrated in many polymer microcapsules, there are currently no studies, to the best of our knowledge, that investigate the utility of GA cross-links in PEMs assembled from bioactive components that must maintain their bioactivity after cross-linking.

Herein, we pursued chemical cross-linking to stabilize MOG/GpG iPEMs and enable loading of Rapa and co-delivery with other cargo. As MOG and GpG components used to assemble iPEMs are naturally compatible for LbL because of their charge, there was no need to introduce other species beyond minute amounts of cross-linker. We demonstrate that cross-linking improves stability of MOG and GpG iPEMs on planar substrates and in injectable capsular iPEMs. Most importantly, we found that cross-linking does not impact the functionality of MOG and GpG. Finally, we show Rapa loading can be achieved in iPEMs and loading efficiency can be modulated in iPEMs as a function of GA concentration and incubation time. These add important functionality to iPEMs to allow loading of small molecule drugs that could pave the way for other combinatorial carrier-free tolerance vaccine platforms.

## 2 Materials and methods

### 2.1 Materials

MOG-R3 peptide (MEVGWYRSPFSRVVLHLYRNGKRRR) was synthesized by Genscript at > 95%purity (Piscataway, NJ). CpG DNA (5’-T*C*C*A*T*G*A*C*G*T*T*C*C*T*G*A*C*G*T*T*T*-3’) and GpG DNA (5’-T*G*A*C*T*G*T*G*A*A*G*G*T*T*A*G*A*G*A*T*G*A*-3’) was purchased from Integrated DNA Technology (Coralville, IA). β-mercaptoethanol, ethylenediaminetetraacetic acid (EDTA), 5x tris-borate-EDTA (TBE) buffer, and bovine serum albumin (BSA) were purchased from Sigma Aldrich (St. Louis, MO). Molecular biology grade water, fetal bovine serum (FBS), 20X phosphate buffered solution (PBS), 20% sodium dodecyl sulfate (SDS), HEPES, and non-essential amino acids were purchased from VWR (Radnor, PA). RPMI-1640 media, SYBR Gold gel stain, l-glutamine, penicillin-streptomycin, Ultrapure Agarose, Brilliant Violet 450 viability stain, and cell proliferation dye eFluor450 were purchased from Thermo Fisher Scientific (Grand Island, NY).

### 2.2 Preparation of cross-linked planar iPEMs

Quartz slides (VWR) were cut into 5 x 25 mm substrates using a diamond-tipped saw (Micro Automation) and cleaned using sequential rinsing in acetone, ethanol, methanol, and water. Washed substrates were modified using an oxygen plasma system (Jupiter III, March) and coated with 20 mM poly(ethylenimine) (PEI, Polysciences, Inc.) with 50 mM NaCl and 5 mM HCl for 5 mins, rinsed for 1 min in milliQ water and coated with 20 mM poly(sodium 4-styrenesulfonate) (SPS, Sigma-Aldrich) with 50 mM NaCl for 5 mins and rinsed after. This process was repeated using a DR3 dipping robot (Riegler & Kirstein GmbH) to achieve ten base layers. Planar substrates were assembled by dipping baselayer coated substrates in 0.5 mg/mL FITC-MOG-R3 for 5 mins, washing twice with milliQ water for 1 min, incubating 0.5 mg/mL GpG for 5 mins, and washing twice with milliQ water for 1 min. This process was repeated to deposit 64 bilayers and substrates were dried under air and stored at room temperature protected from light. Cross-linking was achieved by incubating substrates in glutaraldehyde (GA) in milliQ water at 5%, 10%, and 20% GA for 5 mins followed by two washes to remove excess GA. To investigate stability, cross-linked coated substrates were incubated in 0.05% SDS and 0.1% SDS and washed twice at indicated time points. Absorbance at 480 nm was measured to detect remaining FITC-MOG-R3 on substrates after incubation and washes. UV-vis spectrophotometry was used to measure absorbance at three locations on at least three separate substrates.

### 2.3 Preparation of immune complexes and characterization of cross-links

Electrostatic assembly of immune complexes on calcium carbonate (CaCO_3_) sacrificial templates have been previously demonstrated. To incorporate cargo, 0.5 mg/mL of MOG-R3 was added to CaCO_3_ cores and shaken at 320 rpm for 3 mins before centrifugation. PBS was used to wash the cores after addition of cargo and solution was saved for subsequent additions. 1 mg/mL of GpG was added to cores after 2 washes and process was repeated to achieve three-bilayers with GpG being the final cargo layer. The CaCO_3_ cores were removed using calcium chelator, 1 M EDTA, pH 6 (Sigma, 03690). After core removal, complexes were centrifuged and washed 2x and stored at 4°C in PBS until further use. Loading of MOG-R3 was determined using micro BCA™ Protein Assay (Thermo Scientific, 23235) and GpG loading was quantified using absorbance (Thermo Scientific, Biomate 3S). Cross-linking density was assessed *via* the Fluoraldehyde™ *o*-Phthaldialdehyde (OPA, Thermo Scientific) assay (Ex/Em = 360 nm/455 nm). Size measurements *via* dynamic light scattering were also performed *via* NanoBrook Omni Particle Sizer.

### 2.4 Gel electrophoresis to determine the stability

Formed iPEMs were incubated in PBS, 0.01%, 0.02% SDS, pH 4 buffer, 50x FBS, and 50% trypsin for 2 hours shaking at 120 rpm. 2% agarose gels were created with UltraPure Agarose in TBE buffer and stained with SYBR™ Gold Nucleic Acid Gel Stain (Thermo Fisher Scientific, S11494). Gel Loading Dye, Purple (6X), no SDS (2 µL) (New England BioLabs, B7025S) and immune complex samples (2 µL) were combined and 2 µL was loaded into the gels and ran for 15 minutes at 100V. Images were acquired on the ProteinSimple FluorChem E System Gel Imager. Quantification of band intensity in gels was achieved using ImageJ gel quantification plug-in.

### 2.5 Toll-like receptor assay to test the integrity of cross-linked complexes

HEK-Blue™ mouse TLR9 cells (*In vivo*gen, hkb-mtlr9) were plated at ~280,000 cells/mL in HEK-Blue detection media (*In vivo*gen, hb-det2) according to manufacturer instructions. mTLR9 cells were treated with media (negative control), 10 µg/mL CpG (positive control), or CpG and 20 µg/mL GpG or CpG and MOG/GpG immune complexes (30, 20, 10, and 5 µg/mL). After incubating for 16 hrs, secreted embryonic alkaline phosphatase (SEAP) secretion was measured by spectrophotometry at 650 nm using a Tecan Spark Multimode Microplate Reader.

### 2.6 DC and T cell co-cultures to assess proliferation

All animal care and experiments were carried out in compliance with federal, state, and local guidelines. Cells were isolated from animals that were cared for using protocols reviewed and approved by the institutional animal care and use committee (IACUC) at University of Maryland, College Park. Primary splenic CD11c+ cells were isolated from naïve 9-week female C57bl/6J mice using CD11c+ magnetic isolation beads (Miltenyi, 130-108-338) according to the manufacturer protocol and as previously conducted. Briefly, isolated spleens were minced and incubated in Spleen Dissociation Medium for 30 mins in 37°C, dissociated using a 16G needle, and 10 mM EDTA was added and incubated for 5 minutes at room temperature. For CD11c positive selection, cells were passed through a 40 µm strainer and suspended in MACs buffer (1x PBS, 5 g/L BSA, 2 mM EDTA) containing CD11c+ magnetic isolation beads and placed on an LS column. Isolated DCs were plated at ~560,000 cells/mL and treated with (5 µg/mL soluble CpG, 50 µg/mL soluble GpG, 2.5 µg/mL soluble MOGR3, or 150, 100, 50, and 25 µg/mL immune complexes) in RPMI 1640 media supplemented with 10% FBS, 2 mM L-glutamine, 1x non-essential amino acids, 10 mM HEPES buffer, 1X penicillin and streptomycin, and 55 µM β-mercaptoethanol at 37°C and 5% CO2. After 24 hours, CD4+ T cells were isolated from the spleens and lymph nodes of 2D2 mice using CD4+ T Cell Negative Selection (StemCell Technologies, 19852). Briefly, spleens and lymph nodes were smashed through a 70 µm cell strainer, mixed with normal rat serum, mouse CD4+ T Cell Isolation Cocktail, and Streptavidin RapidSpheres before separation *via* an EasySep Magnet (StemCell Technologies, 18001). Isolated CD4+ T cells were labelled with eFluor450 and added to DC cultures at a 3:1 T cells to DC ratio. Following 72 hours in culture, cells were isolated and stained with fixable NIR Live Dead (Thermo Fisher, L34975). Proliferation was measured using a BD FACS Celesta on the BD FACSDiva™ software and data was analyzed with FlowJo v.10.7 (TreeStar, Ashland, OR). Gating was achieved on single cells by using forward-scatter height versus area (FSC-H vs. FSC-A).

### 2.7 Rapamycin loading and characterization

To load immune complexes, Rapamycin (Rapa, 2 mg/mL in methanol) was added to the complexes while on CaCO_3_ cores. Samples were purified *via* two rounds of centrifugation to remove any aggregated free drug (2 mins at 500 g’s). Core removal was conducted with 1 M EDTA, pH 6 and washed twice. Final solutions of purified cross-linked immune complexes were dissolved in acetonitrile and analyzed using the Waters Alliance™ High Performance Liquid Chromatography (HPLC) system (Waters, e2695). Herein, a mobile phase consisting of A) HPLC grade water + 0.065% trifluoroacetic acid (TFA) and B) HPLC grade acetonitrile + 0.05% TFA was used, and drug elution was monitored using UV/Vis detection at 280 nm (Waters, 2489). Flow conditions were set at 0.1 mL/min with a gradient elution (0 – 0.01 min 5%, 10.40 – 10.42 min 65% B, 10.42 – 12.92 min 95% B, 12.92-29.18 min 5% B). The separation was performed on a Xbridge™ BEH 300 column (50 mm x 2.1 mm, 3.5 µm particle size). The acquired data was analyzed using Waters^®^ Empower^®^ 3 software. Loading capacity was calculated as Rapamycin (mg)/complex (mg).

### 2.8 Statistical analysis

Prism software (GraphPad Version 6.0) was used for all statistical analysis. Multiple comparisons were performed using one-way or two-way ANOVA followed by Tukey’s *post-hoc* analysis or Dunnett’s *post-hoc* analysis to determine significance (p-values are noted in figure legends).

## 3 Results

### 3.1 Glutaraldehyde mediated cross-links stabilize iPEMs on planar substrates

**Figure 1:**
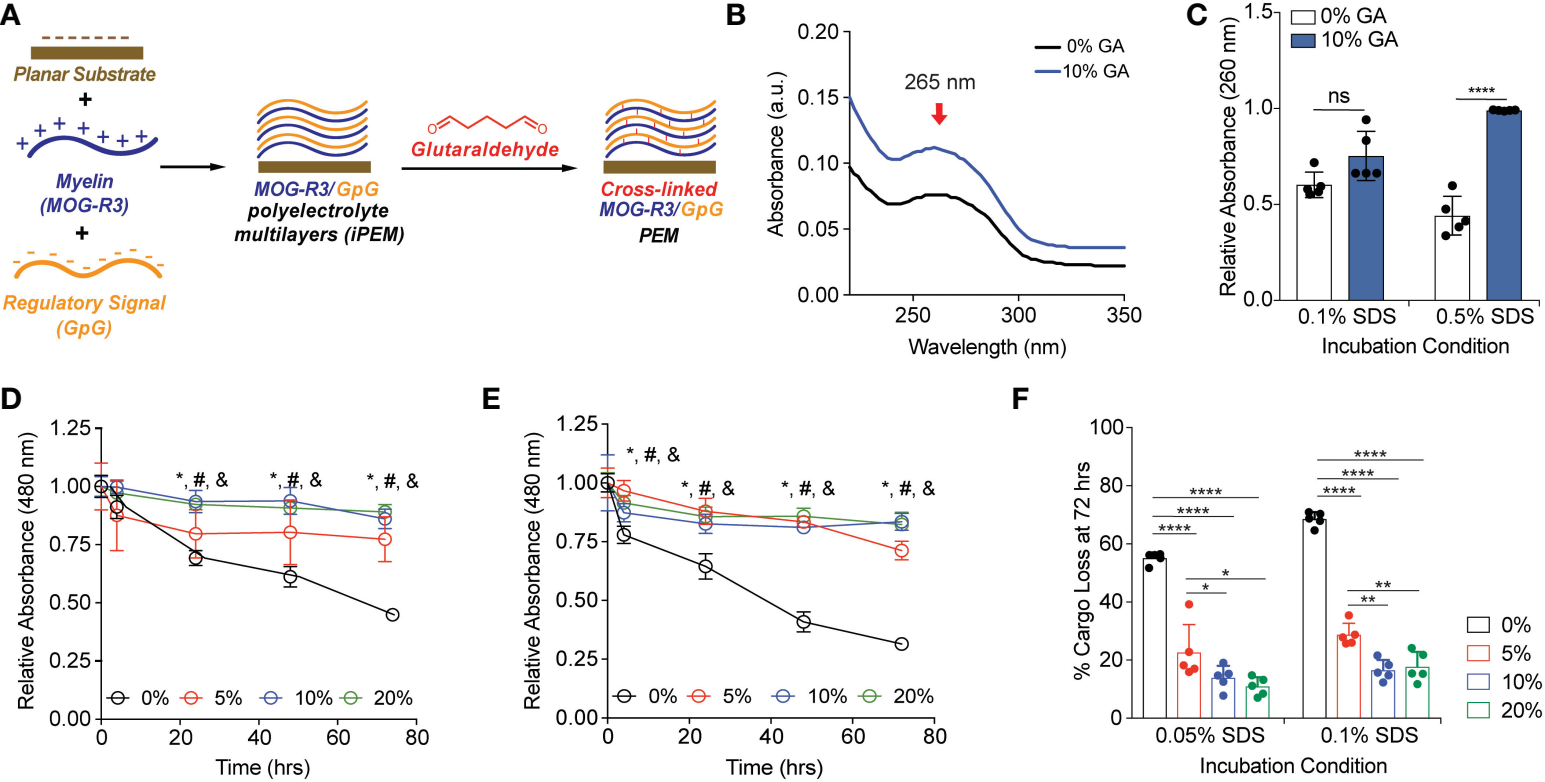
Glutaraldehyde mediated cross-links between MOG-R3/GpG biomolecular layers on planar substrates protect against delamination of the cargo in an anionic surfactant. **(A)** Schematic view of layer-by-layer electrostatic assembly of myelin self-antigen (MOG-R3) and TLR9 antagonist (GpG) on planar substrate, followed by GA mediated crosslinking. **(B)** UV-vis spectra of MOG/GpG planar substrates cross-linked with 10% GA solution. **(C)** Spectrophotometric analysis of planar substrates incubated in various SDS conditions for 15 minutes. Data was normalized to t = 0. Statistical analysis performed using two-way ANOVA comparing 0% GA control to 10% GA in each incubation condition followed by Sidak’s multiple comparisons tests. Data represents mean ± standard deviation (n = 5). ns, not significant. **(D)** Spectrophotometric analysis of planar substrates incubated in 0.05% SDS. **(E)** Spectrophotometric analysis of planar substrates incubated in 0.1% SDS. Data was normalized to t = 0. Statistical analysis performed using one-way ANOVA comparing 0% GA control to crosslinked substrates at a given time point followed by Dunnett’s multiple comparisons tests. Data represents mean ± standard deviation (n = 5). *p < 0.05 between 0% and 5% glutaraldehyde, # p < 0.01 between 0% and 10% GA, and & p < 0.01 between 0% GA and 20% GA. **(F)** Quantification of cargo loss at 72 hrs in 0.05% and 0.1% SDS. Data represents mean ± standard deviation (n = 5). ****p < 0.001% between 0% GA and 5, 10, and 20% GA substrates.

To test our hypothesis that GA can create covalent cross-links to stabilize the multilayers between MOG and GpG iPEMs, we first assembled 64-bilayers of these components on quartz substrates using electrostatic interactions followed by covalent cross-linking (10, 11, 14–16) (**Figure 1A**). GA is a linear, bi-functional cross-linking agent used in a range of applications (27). It reacts rapidly with amines, thiols, phenols, and imidazole functional sites on proteins and peptides to generate chemically stable cross- links (28). We used a characteristic absorbance peak at 265 nm to identify pH-stable pyridinium compound formation or production of pH-responsive Schiff base (27, 29, 30) (**Figure 1B**). Since non-crosslinked iPEMs are assembled through electrostatic interactions that can be readily disrupted in distinct conditions, we next tested if cross-linking can be adapted to stabilize iPEM cargo (i.e., antigen, oligonucleotide) from disassembling in the presence of a strong anionic surfactant, SDS. Absorbance at 260 nm was used to monitor remaining GpG on the planar substrates after multiple washing steps. In 0.1% SDS, the relative absorbance of GpG was not significantly different between non-cross-linked and 10% GA substrates, suggesting similar retention of GpG after multiple washes (**Figure 1C**). Excitingly, at higher SDS concentrations (0.5% SDS), we found that cross-linking protected the cargo from being disassembled, as observed by a decrease in the relative absorbance of 0% GA substrates compared to 10% GA substrates (**Figure 1C**). Next, we exposed MOG/GpG iPEMs to distinct GA concentrations; this allowed us to test if modulating cross-linking density would alter stability and disassembly. In kinetics studies we found that non-cross-linked substrates were disassembled at a faster rate than cross-linked MOG/GpG iPEMs (**Figures 1D, E**). In the 0.05% SDS condition, the loss of cargo signal was similar for all GA cross-linking concentrations, and these were all significantly higher than the non-cross-linked conditions (**Figure 1D**). Of note, at the higher 0.1% SDS concentration, 5% GA-treated substrates disassembled faster than 10% and 20% GA-treated substrates at 72 hrs (**Figure 1E**); this suggests that GA concentration can be used to impart tunable stability in the presence of competing forces, such as strong anionic solvents. The quantitative values for all conditions are shown in **Table S1**. At the end of the 0.05% SDS study, 55 ± 2% cargo loss was observed for non-cross-linked iPEM- coated substrates, while cross-linked substrates lost < 25% of cargo (**Figure 1F**). Similarly, for the more stringent challenge of 0.1% SDS, 69 ± 2% loss was observed in non-cross-linked iPEM-coated substrates, while < 30% of cargo was disassembled from cross-linked iPEM design (**Figure 1F**). These studies confirm our hypothesis that exposure to GA stabilizes iPEMs due to cross-link formation. This also suggests the level of GA, and thus cross-linking density, could be potentially used to control the release kinetics of iPEMs on planar substrates, or to load small molecular cargo when iPEMs are synthesized in capsular form.

### 3.2 Cross-linking density can be tuned in iPEM capsules using GA

**Figure 2:**
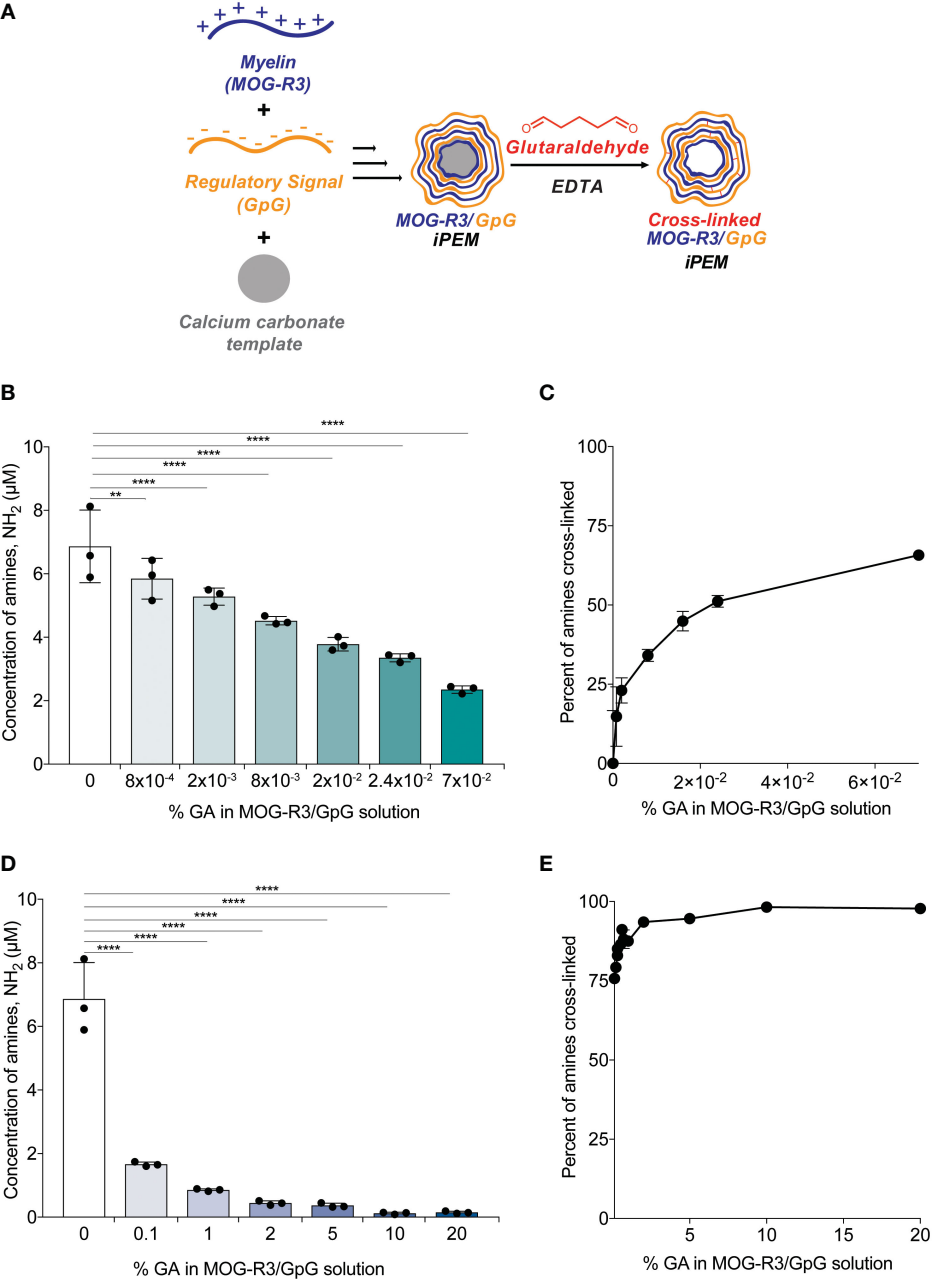
Cross-linking density can be modulated by changing the percent of glutaraldehyde in solution. **(A)** Schematic view of layer-by-layer electrostatic assembly of myelin self-antigen (MOG-R3) and TLR9 antagonist (GpG) on sacrificial template, followed by glutaraldehyde mediated crosslinking. **(B–E)** Fluoraldehyde assay showing degree of crosslinking after 5 minutes of GA incubation. Data represents mean ± standard deviation. **p < 0.01, ****p < 0.001 versus 0% GA iPEMs using one-way ANOVA and Dunnett’s multiple comparisons test.

Since we demonstrated that stable cross-links could be formed on planar substrates (**Figure 1**), we next tested if GA cross-linking can be controlled in capsular MOG/GpG iPEMs. iPEMs were assembled on sacrificial CaCO_3_ templates, then defined GA concentrations were added prior to removal of the template using a EDTA (**Figure 2A**). We used an OPA fluoraldehyde assay to directly measure the concentration of primary amines in the capsules after GA incubation; thus, a reduction in primary amine signal is expected due to amine condensation by GA. When low levels of GA (8x10^-4^%) were added to iPEMs, a significant loss in amine signal was observed, correlating to 15 ± 9% cross-linking; this suggests immediate cross-linking at low GA concentrations (**Figures 2B, C**). Incubating iPEMs at a 10-fold higher GA concentration (8x10^-3^%) resulted in further loss of amine signal (**Figure 2B**), correlating to 34 ± 9% cross-linking (**Figure 2C**). The sensitivity of GA concentration in cross-linking iPEMs was especially noticeable as 2x10^-2^% GA and 2.4x10^-2^% GA, which resulted in 45 ± 3% cross- linking and 51 ± 2% cross-linking, respectively (**Figures 2B, C**). At 7x10^-2^% GA, 66 ± 2% cross-linking had been achieved, further demonstrating the cross-linking efficiency of GA (**Figure 2C**). Next, we tested GA concentrations at 0.1% GA and above (**Figure 2D**). We showed that at 0.1% GA resulted in 76 ± 2% cross-linking and 1% GA resulted in 86 ± 2% (**Figure 2E**). As GA concentration further increased to 2% GA, 94 ± 1% cross-linking was observed, while 20% GA peaked at 98 ± 1% crosslinking; this is essentially complete cross-linking (**Figure 2E**). Notably, changing GA concentrations did not significantly impact the morphology or size of iPEMs (**Figure S1**). Since GA incubation time has been shown to increase cross-linking density in other studies (31), we next tested GA incubation for 5 minutes and 30 minutes. After incubating iPEMs for 30 minutes, we found that longer incubation of iPEMs with GA resulted in higher degrees of cross-linking in 8x10^-4^% and 0.1% GA treated complexes (**Figure S2**). Altogether, this study found that adjusting GA concentration and incubation time controls cross-linking density in iPEMs.

### 3.3 Cross-linking improves the stability of iPEM capsules in conditions of physiological stress

**Figure 3:**
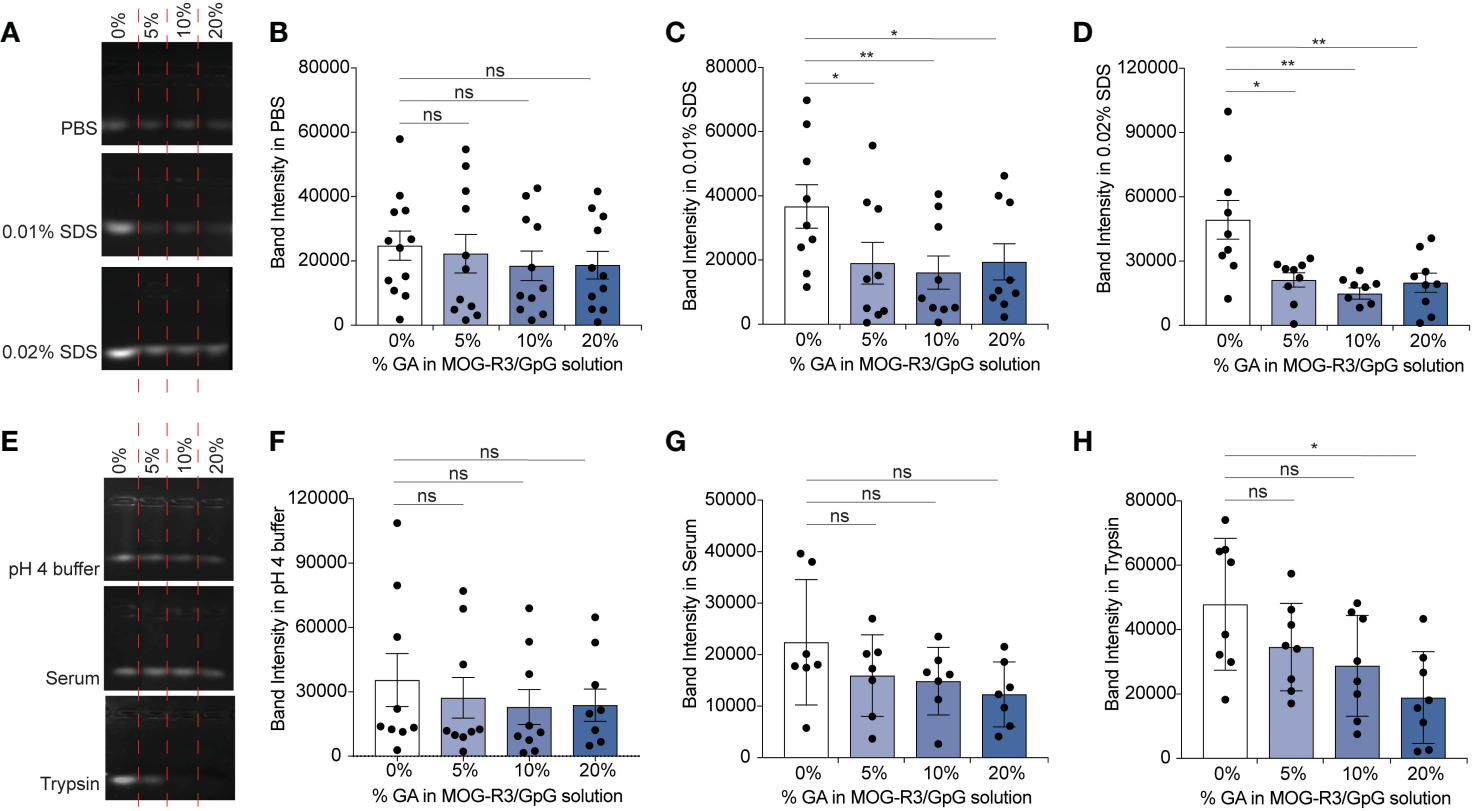
GpG in iPEM capsules is maintained in capsular form when cross-linked and incubated in various conditions. **(A)** Representative agarose gel of non-cross-linked and cross-linked MOG-R3/GpG iPEMs incubated in PBS, 0.01% SDS, 0.02% SDS and **(B–D)** quantification of the band intensities observed for each condition. **(E)** Agarose gel of non-cross-linked and cross-linked MOG-R3/GpG iPEMs incubated in pH 3-4 buffer, fetal bovine serum, and Trypsin and **(F–H)** quantification of the band intensities observed for each condition. Data represents mean ± standard error mean from multiple iPEM batches (N = 8-12). Statistical analysis performed using one-way ANOVA followed by Dunnett’s multiple comparisons between 0% iPEMs and cross-linked iPEMs. *p < 0.05, **p < 0.01, ns, not significant.

As GA mediated cross-linking produces covalent bonds between amine-containing cargo, we hypothesized that GA would stabilize iPEM capsules in specific conditions of physiological stress. Gel electrophoresis was used to monitor the presence of unbound GpG in iPEMs by using a fluorescent nucleic acid stain that intercalates with nucleic acids to show a strong fluorescent band for unbound GpG in the gels (**Figures 3A, E**). In this assay, only unbound GpG migrates through the gel and can be visualized by the dye (32), while complexed iPEMs remains in the well without signal. Since iPEMs are prepared in PBS, we determined the baseline signal of unbound GpG when iPEMs are cross-linked. In PBS, the band intensity of unbound GpG in non-cross-linked and cross-linked complexes were statistically equivalent, although the bands for non-cross-linked iPEMs appeared slightly brighter (**Figures 3A, B**); this suggests cross-linking maintains iPEM complexes more effectively than electrostatic interactions in non-cross-linked iPEMs. In 0.01% and 0.02% SDS, iPEMs cross-linked with 5%, 10%, and 20% GA exhibited weaker bands compared to non-cross-linked iPEMs (**Figures 3A, C, D**). This finding suggests that although SDS can dissociate cross-linked iPEMs, it is more effective at disassembling non-cross-linked iPEMs built solely on electrostatic interactions. Thus, covalent cross- links can be used to maintain the stability of iPEMs.

Next, we incubated the iPEMs in pH 4 buffer to test if pH responsive Schiff bases were formed in iPEMs, interactions that could potentially be disrupted upon endocytic uptake during similar pH changes (**Figure 3E**). In pH 4 buffer, cross-linked iPEMs exhibited comparable band intensities to non- cross-linked designs, suggesting that bonds formed in iPEMs were not pH responsive (**Figures 3E, F**). Since in biological conditions nucleic acids such as GpG are subjected to potential degradation by nucleases, we next tested the stability of the cross-links in serum, which contains a range of nucleases. We found similar band intensities between non-cross-linked iPEMs and cross-linked iPEMs tested (**Figures 3E, G**). Finally, we tested iPEMs in trypsin to test if cross-linking protects GpG against serine proteases involved in digestion. We found that the band intensity for non-cross-linked iPEMs was significantly higher than for cross-linked designs, suggesting protection of GpG from digestive enzymes after cross-linking (**Figures 3E, H**). Overall, these results confirmed that cross-linking improves the stability of iPEMs in various conditions and suggests that a key feature of cross-linking could be to improve cargo protection during administration. Furthermore, by demonstrating that cross-linking improves stability in degradation enzymes, these studies suggest potential routes to tune iPEM release for autoimmune diseases involving digestive organs, such as celiac disease.

### 3.4 TLR antagonists in cross-linked iPEMs retain the ability to blunt TLR9 signaling

**Figure 4:**
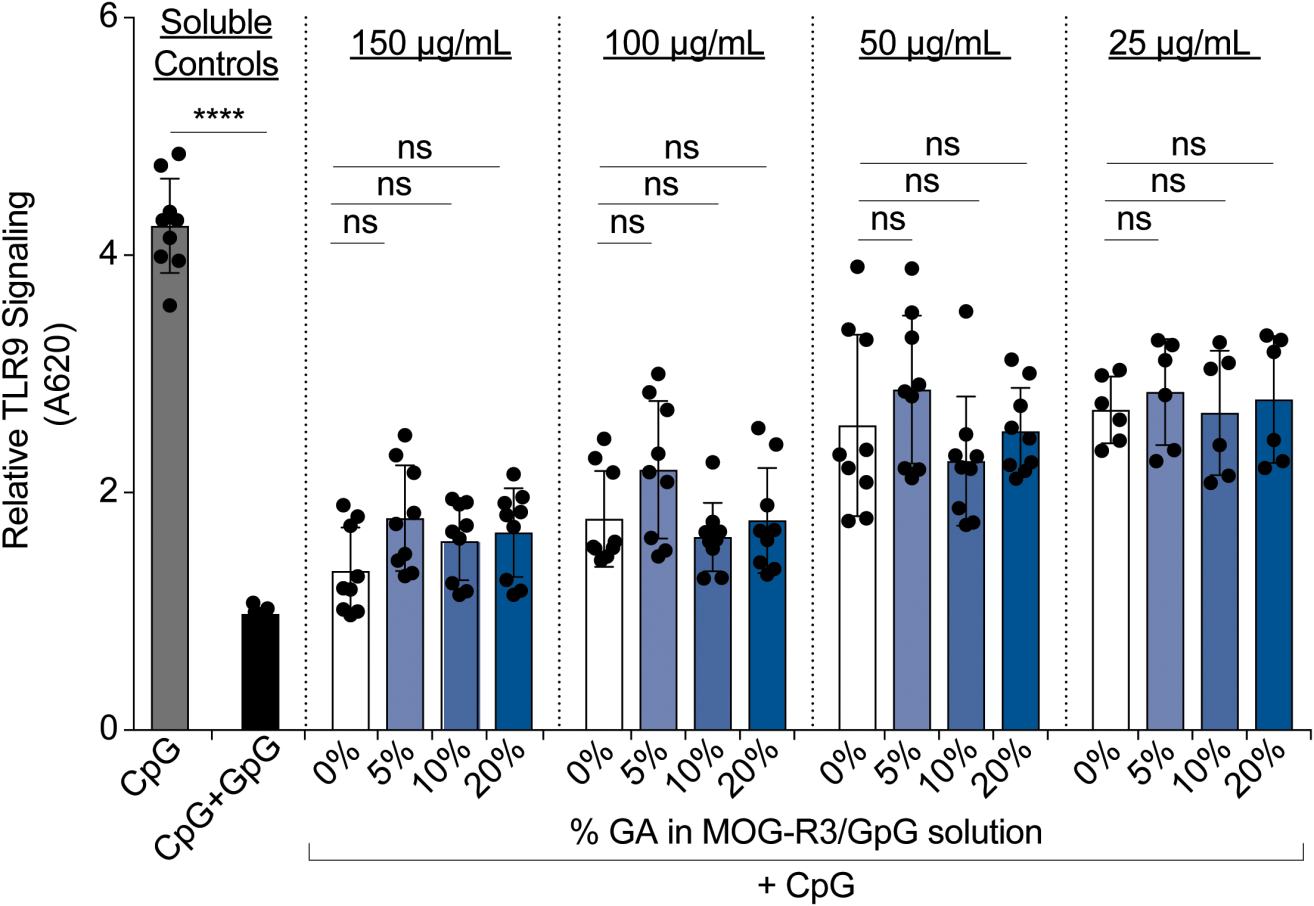
Cross-linked iPEMs maintain their ability to blunt TLR9 signaling. Relative TLR9 signal after 14-hour incubation with iPEMs. Data represents mean ± standard error mean of two to three independent experiments completed in triplicates. Statistical comparisons performed using two-way ANOVA followed by Dunnett’s multiple comparisons test between non-cross-linked and cross-linked iPEMs. Ns, not significant. Unpaired t-tests were used to compare soluble controls. ****p < 0.0001.

Unlike other PEMs reported using synthetic materials, iPEMs are composed entirely of bioactive immune cues that must maintain their functionality upon cross-linking. Therefore, we next investigated if GpG in iPEMs maintain the ability to restrain TLR9 signaling. TLR9 is an endosomal TLR that senses pathogen-associated nucleic acids rich in cytosine and guanine residues such as CpG to trigger inflammatory responses. GpG binds TLR9 with similar binding affinity as CpG (33) and competes with CpG binding to restrain TLR9 signaling (19). In these studies, we treated TLR9 reporter cells with CpG to activate TLR9, then added cross-linked iPEMs to test if TLR9 signaling could be blunted (**Figure 4**). As expected, adding soluble GpG to compete with CpG, resulted in significant reduction of TLR9 signaling (**Figure 4**). When TLR9 reporter cells were treated with soluble CpG and cross-linked iPEMs at distinct concentrations of total iPEM, the TLR9 signaling was similarly restrained between cross- linked and non-cross-linked iPEM controls (**Figure 4**). This finding indicated that GpG cross-linked in iPEMs retain their ability restrain CpG-driven inflammation, while also offering improved stability (**Figure 3**).

### 3.5 Cross-linked iPEMs maintain their ability to engage cognate T cell receptor

**Figure 5:**
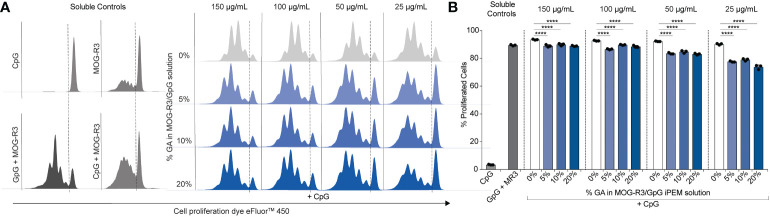
Cross-linked iPEMs induce 2D2 T cell proliferation. **(A)** Representative histograms of T cells analyzed by flow cytometry. Dotted line separates undivided cells from divided cells. **(B)** Percent of live proliferated CD4+ T cells in culture after treatment with iPEMs. Data represents mean ± standard deviation. Statistical analysis performed using two-way ANOVA followed by Dunnett’s test comparing non-cross-linked iPEMs to cross-linked groups. ****p < 0.0001.

After observing that GpG activity in cross-linked iPEMs was maintained (**Figure 4**), we tested if MOG also maintained its ability to engage its cognate receptor on primary myelin-specific T cells. Recognition of peptide in major histocompatibility complex (MHC) molecules on DCs by T cell receptors on antigen-specific T cells is a key event for T cell activation. In this study, we used MOG- specific CD4^+^ T cells from 2D2 transgenic mice, in which all T cells express T cell receptors specific for MOG peptide. Thus, when these T cells encounter DCs presenting MOG, the cognate antigen engagement results in T cell proliferation. Therefore, if MOG in cross-linked iPEMs is available for intracellular processing and loading onto MHC-II to bind cognate receptors on 2D2 T cells, we would expect comparable proliferation relative to non-cross-linked iPEMs. DCs were treated with iPEMs for 24 hours and co-cultured with MOG-specific T cells for 72 hours. As expected, when co-cultures were treated with soluble CpG without MOG, T cells did not proliferate; this is indicated by a high level of fluorescence at the end of the study in the CpG soluble control sample (**Figure 5A**). Conversely, in a control treated with soluble GpG and MOG-R3, >80% of T cells proliferated; this is indicated by successive dilution of the fluorescence dye used in this assay during each round of MOG-induced T cell division (**Figure 5A**).

When co-cultures were treated with cross-linked iPEMs using a range of iPEM doses, up to 5 division were observed at the lowest iPEM dose tested, and up to 4 divisions were observed for the highest dose tested, indicating that MOG is presented through MHC molecules to the antigen-specific T cells (**Figure 5**). Although cross-linking did not hinder T cell proliferation altogether, comparisons to non-cross- linked iPEMs at each concentration tested revealed that the percent of proliferated cells were reduced in cultures treated with cross-linked iPEMs. This finding suggests three possibilities, 1) not all MOG peptides in cross-linked iPEMs are presented in MHC molecules and available to bind to available T cell receptors, 2) cross-linking affects the binding affinity between MOG presented in MHC molecules and T cell receptor, or 3) cross-linking delays receptor binding. Since we did not directly test binding affinity, it is difficult to make definitive conclusions regarding the specific mechanism. However, we found that after co-culturing T cells with DCs for 96 h, the percent of proliferated cells were the same for all groups (**Figure S3**). Based on this finding, it is likely that cross-linking modestly delays MOG presentation in MHC molecules and engagement of the T cell receptor, altering the kinetics. Nonetheless, our data showing that T cells proliferate in across cross-linking density suggests that the function of MOG is not impacted by GA.

### 3.6 iPEMs load Rapamycin despite cross-linking density

Since we demonstrated iPEMs assembled from MOG and GpG can be cross-linked to improve stability without impacting the function of the components, we next aimed to expand the capabilities of the iPEMs through drug loading of small molecule immunomodulatory cue (e.g., Rapa). Loading bioactive drugs in conventional synthetic PEMs can be accomplished by incorporating drug before layer-by- layer deposition or through adding drug post-layer-by-layer build into multilayers (34). Rapa is a clinically important immunosuppressant used for kidney transplant patients. More recently, Rapa has been used as an immunomodulatory cue in preclinical MS studies owing to its neuroprotective, neurotrophic, and anti-aging properties (24). Importantly, our group has previously shown that co-delivery of Rapa with peptides bias T cells towards regulatory phenotypes (13, 28, 35). Adding a small molecular drug loading capability to iPEMs could enable delivery of multiple classes of modulatory cues in a simple, carrier- free platform that juxtaposes self-peptide, innate regulatory ligands (i.e., CpG), and adaptive cues (i.e., Rapa).

**Figure 6:**
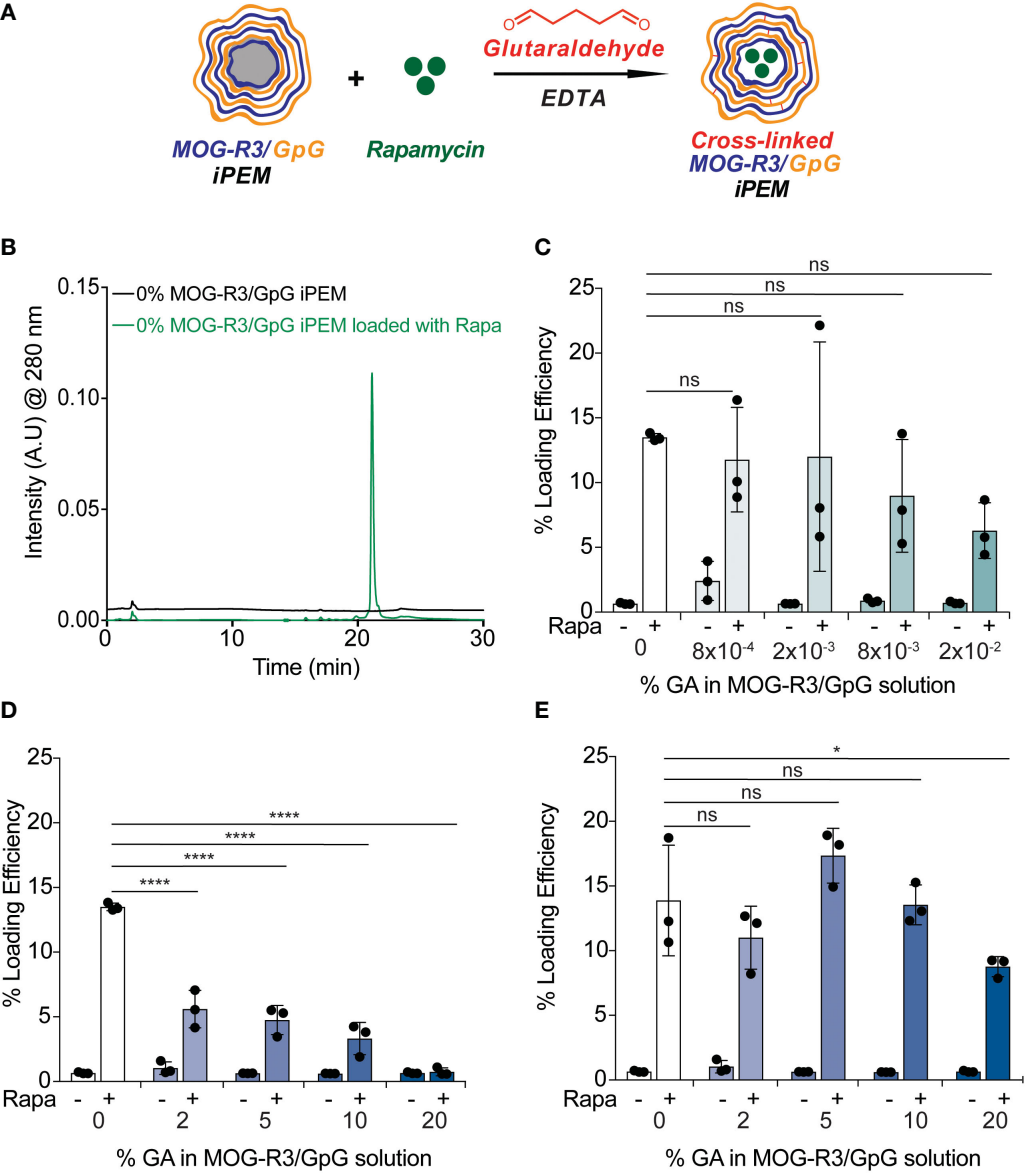
Rapamycin loading is achieved in non-cross-linked and cross-linked iPEMs. **(A)** Schematic view of MOG-R3/GpG capsules loaded with Rapamycin and crosslinked with glutaraldehyde. **(B)** High performance liquid chromatography trace indicating Rapa loading in non-cross-linked iPEMs. **(C, D)** Loading efficiency = mg drug loaded/mg drug initial of iPEMs loaded for 5 minutes with Rapa. **(E)** Loading efficiency of iPEMs loaded for 2 hrs with Rapa. Data represents (n = 3 ± standard deviation). Two-way ANOVA followed by Tukey’s multiple comparisons test used to assess statistical differences between samples. *p < 0.05, ****p < 0.0001, ns, not significant.

Owing to the potential of targeting both innate and adaptive immune cells through co-delivery of Rapa, MOG, and GpG, we first tested if Rapa can be loaded into iPEMs (**Figure 6A**). Using HPLC, we found that Rapa loading could be detected at 280 nm in iPEMs incubated with Rapa, while iPEMs not containing Rapa did not (**Figure 6B**). Excitingly, this correlated to a loading efficiency of ~ 14 ± 0.3%; this represents the amount of Rapa loaded per the initial Rapa amount used in the loading feed in iPEMs without cross-linking (**Figure 6C**). We also tested if cross-linking can be used to improve Rapa loading into iPEMs. Previous studies investigating drug loading into PEM films found that higher amounts of drug were loaded in conventional synthetic PEMs without cross-linking, relative to cross-linked counterparts. In other studies, cross-linking improved drug loading (31, 36, 37). To reveal the role of cross- linking in this new system where PEMs are assembled entirely from immune cues, we held Rapa concentration and added this drug to assembled iPEMs on templates prior to cross-linking and core removal (**Figure 6A**). After incubating Rapa and iPEMs for 5 minutes, we found that iPEMs cross-linked with 8x10^-4^, 2x10^-3^, 8x10^-3^, and 2x10^-2^ GA exhibited comparable Rapa loading to non-cross-linked iPEMs (**Figure 6C**). In contrast, iPEMs cross-linked with 2%, 5%, 10%, and 20% GA exhibited statistically reduced Rapa loading compared to non-cross-linked iPEMs (**Figure 6D**). We hypothesized that increased cross-links limit diffusion of Rapa, thus we next tested if incubating Rapa and iPEMs for 2 hrs would improve Rapa loading in high GA iPEMs. Interestingly, we found that increasing Rapa incubation time to 2 hrs resulted in comparable loading between non-cross-linked iPEMs and 2%, 5%, and 10% GA iPEMs (**Figure 6E**). Notably, increasing the incubation time of Rapa and 20% GA iPEMs resulted in improved loading efficiency of ~10% compared to < 1% loading efficiency observed when iPEMs were incubated for 5 minutes (**Figures 6D, E**). Altogether, we show for the first time that iPEMs can be loaded with a small molecule immunomodulator with and without addition of a cross-linker. However, we showed cross-linking provides improved stability and a route to regulate release or delivery depending on environmental cues. Thus, incorporating these small molecule drugs further broadens the applicability of iPEMs to be attack multiple pathways in future autoimmune therapy application.

## 4 Discussion

The data above show incorporating chemical cross-links into precisely controlled self-assembled immune cues - self-antigen and toll-like receptor antagonist - does not hinder the functional properties of the material and loading of additional classes of therapeutics can be achieved. It is well-established that APCs present three signals – antigen, costimulatory cues, cytokines –to drive T cell differentiation towards regulatory or inflammatory phenotypes. In T-cell-mediated autoimmune diseases such as MS, pre-clinical studies delivering self-antigens have emerged as novel therapies with limited success in clinical translation (38). The most promising pre-clinical studies delivering self-antigens for MS exploit the co-delivery of regulatory cues to achieve antigen-specific responses. Toll-like receptors recognize pathogen associated molecular patterns (PAMPs) on foreign pathogens and are elevated in human and preclinical MS models (39). In one study, repeated doses of toll-like receptor antagonist, GpG, resulted in modest efficacy that was not long-lasting in preclinical MS model, experimental autoimmune encephalomyelitis (EAE) (40). Our previous work shows that co-delivery of MOG and GpG is required to generate immune tolerance in established pre-clinical MS, thereby highlighting the advantage of combinatorial delivery of these distinct cues. We adapted covalent cross-linking into iPEMs to strengthen the interactions of MOG and GpG and to protect the iPEMs from undesired disassembly in low pH and high salt conditions. This modification of the iPEM capsule was necessary for ensuring that MOG and GpG were co-delivered to APCs for antigen presentation and TLR9 antagonism in the endosomes. In similar studies investigating GA-mediated cross-linking in PEMs, pH-responsive Schiff bases were formed, and improved mechanical strength was observed (30). In our system, we used gel electrophoresis to assess the interactions between MOG and GpG. We found lower fluorescent signal in cross-linked iPEMs compared to non-cross-linked iPEMs, suggesting that we had strengthened the interactions between the components with GA cross-linking.

Interestingly, we show that covalent cross-linking of iPEMs does not hinder the ability of GpG to blunt TLR9 signaling and MOG to induce antigen-specific T-cell proliferation. This finding is important because it is contrary to the gel electrophoresis data that suggest strong interactions between GpG and MOG after cross-linking. This disagreement between the gel electrophoresis data and the functional assays highlights a potential limitation with using simplified systems to emulate complex conditions in cell-culture media. Nonetheless, our findings showing that iPEMs promote antigen-specific T cell proliferation are crucial because it suggests that cross-linking the residues on MOG does not compromise peptide processing and presentation by APCs or recognition by T cell receptors.

It is critical that MOG and GpG are functional after cross-linking. This is because prior work shows that both components are required to reduce disease severity in EAE, to promote regulatory T cells, and to decrease inflammatory transcripts (19). Our present work confirming that cross-linked and non-cross-linked iPEMs comparingly blunt TLR9 signaling suggests that downstream inflammatory processes are also comparably downregulated, although we did not explicitly test that in this study. These comparable results paired with the expansion of myelin-specific T cells suggest that cross-linked iPEMs could be used to promote regulatory T cells to treat MS, as we have demonstrated in prior work.

Beyond examining the impact of cross-linking on iPEM component function, we showed for the first time that Rapamycin could be loaded into iPEMs. Rapamycin loading provides an opportunity to arrest T-cell expansion and promote regulatory T cells as demonstrated by many others (13, 20–23). In the context of treating MS and other autoimmune diseases, we expect that the co-delivery of MOG, GpG, and Rapamycin will robustly downregulate proinflammatory cues to drive naïve T cells towards regulatory phenotypes.

## Data availability statement

The raw data supporting the conclusions of this article will be made available by the authors, without undue reservation.

## Ethics statement

Cells were isolated from animals that were cared for using protocols reviewed and approved by the institutional animal care and use committee (IACUC) at University of Maryland, College Park.

## Author contributions

MAF and CMJ designed the experiments presented herein and drafted the initial version of the document with figures. MAF conducted the studies and analyzed the data. CMJ provided input on manuscript design and scope. All authors contributed to the article and approved the submitted version.
