# Supplementary material for: Enhancing the functionality of self-assembled immune signals using chemical crosslinks
Source: Front Immunol. 2023 Feb 6;14:1079910. doi: 10.3389/fimmu.2023.1079910 (PMC9940312; doi:10.3389/fimmu.2023.1079910)
Supplement: Supplementary file 1 [file DataSheet_1.docx]

**Enhancing the Functionality of Self-Assembled Immune Signals Using Chemical Crosslinks**

Marian Ackun-Farmmer^1^ and Christopher M. Jewell^1-5^

^1^ Fischell Department of Bioengineering, University of Maryland

College Park, MD, 20742, USA

^2^ US Department of Veterans Affairs, VA Maryland Health Care System

Baltimore, MD, 21201, USA

^3^ Robert E. Fischell Institute for Biomedical Devices

College Park, MD, 20742, USA

^4^ Department of Microbiology and Immunology, University of Maryland Medical School

Baltimore, MD, 21201, USA

^5^ Marlene and Stewart Greenebaum Cancer Center

Baltimore, MD, 21201, USA

*** Correspondence:** Christopher M. Jewell, Ph.D.
 [cmjewell@umd.edu](mailto:cmjewell@umd.edu)

**Keywords:** layer-by-layer self-assembly, polyelectrolyte multilayer, cross-linking, microparticle and nanoparticle, vaccine, and immunotherapy

Abstract

Multiple sclerosis (MS) is an autoimmune disease that develops when dysfunctional autoreactive lymphocytes attack the myelin sheath in the central nervous system. There are no cures for MS, and existing treatments are associated with unwanted side effects. One approach for treating MS is presenting distinct immune signals (i.e., self-antigen and immunomodulatory cues) to innate and adaptive immune cells to engage multiple signaling pathways involved in MS. We previously developed immune polyelectrolyte multilayer (iPEM) complexes built through layer-by-layer deposition of self-antigen - myelin oligodendrocyte glycoprotein (MOG) - and toll-like receptor antagonist, GpG to treat MS. Here, glutaraldehyde-mediated stable cross-links were integrated into iPEMs to load multiple classes of therapeutics. These cross-linked iPEMs maintain their immunological features, including the ability of GpG to blunt toll like-receptor 9 signaling and MOG to expand T cells expressing myelin-specific T cell receptors. Lastly, we show that these functional assemblies can be loaded with a critical class of drug – mTOR inhibitors - associated with inducing regulatory T cells. These studies demonstrate the ability to incorporate small molecule drugs in reinforced self-assembled immune signals juxtaposed at high densities. This precision technology contributes new technologies that could drive antigen-specific immune response by simultaneously modulating innate and adaptive immunity.


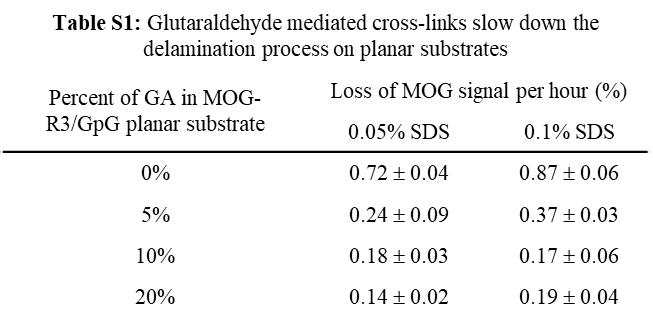


**
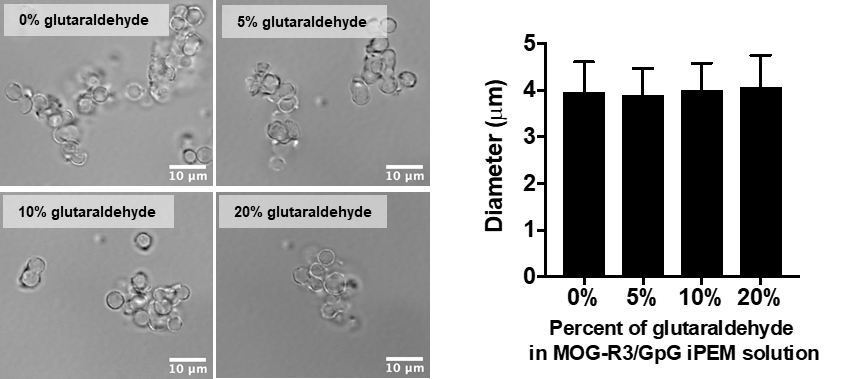
**

**Figure S1: Crosslinked iPEMs maintain their physiochemical and functional properties.** (A) Light microscopy of iPEMs. Scale bar = 10 μm. (D) Diameters of iPEMs.


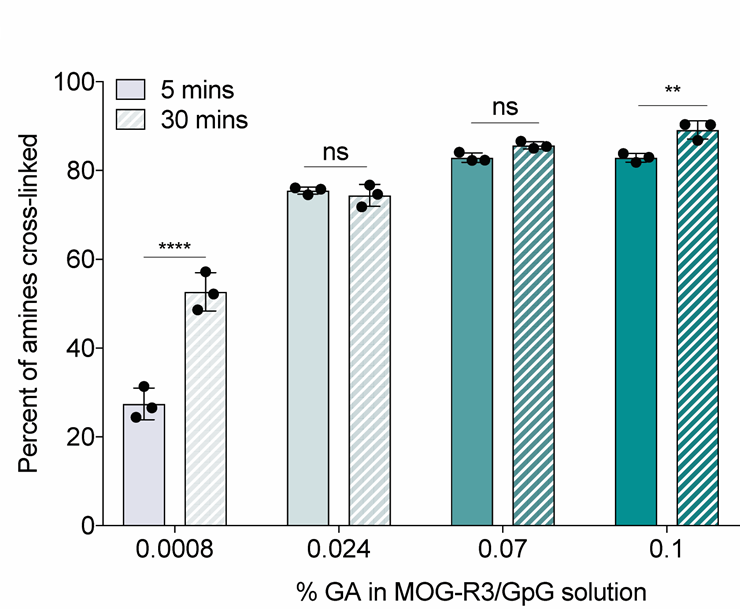


**Figure S2: Longer GA incubation times increase cross-linking density.** Fluoraldehyde assay showing degree of crosslinking after 5 and 30 minutes of GA incubation. Data represents mean ± standard deviation. *** p < 0.01 comparing 5 mins and 30 min incubation times using two-way ANOVA and Sidak’s multiple comparisons test.


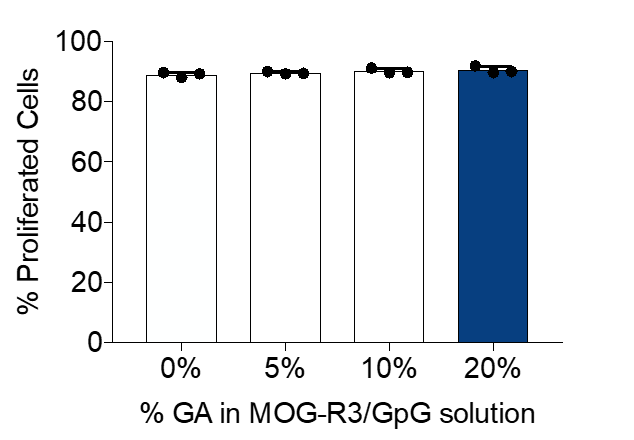


**Figure S3: The percent of proliferated cells are similar in DC/T cell co-cultures after 96 hours of incubation.** Percent of live proliferated CD4+ T cells in culture after treatment with iPEMs. Data represents mean ± standard deviation. No statistical differences were noted between groups using one-way ANOVA.
